# Functional Studies and In Silico Analyses to Evaluate Non-Coding Variants in Inherited Cardiomyopathies

**DOI:** 10.3390/ijms17111883

**Published:** 2016-11-10

**Authors:** Giulia Frisso, Nicola Detta, Pamela Coppola, Cristina Mazzaccara, Maria Rosaria Pricolo, Antonio D’Onofrio, Giuseppe Limongelli, Raffaele Calabrò, Francesco Salvatore

**Affiliations:** 1 CEINGE-Biotecnologie Avanzate s.c.a r.l., 80145 Napoli, Italy; gfrisso@unina.it (G.F.); detta@ceinge.unina.it (N.D.); coppolap@ceinge.unina.it (P.C.); cristina.mazzaccara@unina.it (C.M.); 2 Dipartimento di Medicina Molecolare e Biotecnologie Mediche, Università di Napoli Federico II, 80131 Napoli, Italy; mariarosaria.pricolo@unina.it; 3 Dipartimento di Cardiologia, Seconda Università degli Studi di Napoli, A.O. Monaldi, Azienda dei Colli, 80131 Napoli, Italy; donofrioant@iol.it (A.D.); limongelligiuseppe@libero.it (G.L.); raffaele.calabro@unina2.it (R.C.); 4 IRCCS-Fondazione SDN, 80143 Napoli, Italy

**Keywords:** minigene, splicing analysis, inherited cardiomyopathies, non-coding variations, intronic mutations, *SCN5A*, *MYBPC3*, *ACTC1*

## Abstract

Point mutations are the most common cause of inherited diseases. Bioinformatics tools can help to predict the pathogenicity of mutations found during genetic screening, but they may work less well in determining the effect of point mutations in non-coding regions. In silico analysis of intronic variants can reveal their impact on the splicing process, but the consequence of a given substitution is generally not predictable. The aim of this study was to functionally test five intronic variants (*MYBPC3*-c.506-2A>C, *MYBPC3*-c.906-7G>T, *MYBPC3*-c.2308+3G>C, *SCN5A*-c.393-5C>A, and *ACTC1*-c.617-7T>C) found in five patients affected by inherited cardiomyopathies in the attempt to verify their pathogenic role. Analysis of the *MYBPC3*-c.506-2A>C mutation in mRNA from the peripheral blood of one of the patients affected by hypertrophic cardiac myopathy revealed the loss of the canonical splice site and the use of an alternative splicing site, which caused the loss of the first seven nucleotides of exon 5 (*MYBPC3*-G169AfsX14). In the other four patients, we generated minigene constructs and transfected them in HEK-293 cells. This minigene approach showed that *MYBPC3*-c.2308+3G>C and *SCN5A*-c.393-5C>A altered pre-mRNA processing, thus resulting in the skipping of one exon. No alterations were found in either *MYBPC3*-c.906-7G>T or *ACTC1*-c.617-7T>C. In conclusion, functional in vitro analysis of the effects of potential splicing mutations can confirm or otherwise the putative pathogenicity of non-coding mutations, and thus help to guide the patient's clinical management and improve genetic counseling in affected families.

## 1. Introduction

Once a new mutation has been identified, the next step is to look for its pathogenicity, which may be assessed at different confidence levels. According to the American College of Medical Genetics and Genomics (ACMG), the most important criteria to establish causality of putative disease-causing mutations are minor allele frequency (MAF), co-segregation, and in silico pathogenicity scores [1,2,3]. Co-segregation of a novel variant with disease within families is useful for variant classification, but may be complicated by the structure of the pedigree, the issue of non-paternity, and by variable penetrance and expression of the disease. Several bioinformatics studies have been performed in the attempt to understand the molecular effects of variations in coding regions and in the consensus sequences of the 3′ and 5′ splice sites (3′ss and 5′ss) [4,5]. In general, no bioinformatics tool achieves complete accuracy and the predicted results are sometimes inconsistent. Moreover, there may be discordance between in silico predictions and in vitro or in vivo functional studies, possibly due to features not considered in the in silico approach [4,6].

A reliable functional assay is generally the best means with which to characterize the biological effect of variants. Ideally, when a genetic variation is located in intronic regions, the patient’s RNA should be analyzed by RT-PCR to verify if the variant potentially involved in pre-mRNA maturation does indeed affect splicing [7,8]. If mRNA cannot be obtained due to the lack of an appropriate tissue sample, a minigene or ectopically expressed mRNA (e.g., cardiac mRNAs extracted from peripheral blood) can be used to experimentally analyze the intronic variations [9,10,11]. In many instances minigene splicing analysis proved to be a sensitive and specific system for the study of presumptive splice mutations [12,13,14].

In this study, we evaluated the effect on the splicing mechanism of five different intronic variations, found in three genes (*SCN5A*, *MYBPC3*, and *ACTC1*) of five patients/families, and probably implicated in the triggering of two inherited cardiomyopathies: Brugada syndrome (BrS) and hypertrophic cardiomyopathy (HCM). Using an integrated approach, we first used the ACMG criteria to establish the pathogenic effects of these variants, and then evaluated their function by mRNA or minigene analysis.

## 2. Results

### 2.1. Clinical Molecular Genetics

In the context of a large genetic screening of about 400 patients from Southern Italy affected by inherited cardiomyopathies associated to sudden death risk, we analyzed a panel of 20 genes implicated in these disorders (Table 1).

We found 136 different mutations, of which 11 intronic variants were in the region of splice sites. We did not analyze non-coding variants already functionally characterized or intronic variants affecting canonic splicing sites when we did not have the patient’s mRNA. Thus, we focused on five non-coding variants found in five independent patients (affected by HCM or BrS) and located in three genes: two novel variants in *MYBPC3* (c.2308+3G>C) and *ACTC1* (c.617-7T>C), and two known, but not functionally characterized, disease-associated variants, one in *MYBPC3* (c.506-2A>C) and the other in *SCN5A* (c.393-5C>A) [15]. Finally, we analyzed the known variant c.906-7G>T in the *MYBPC3* gene, which had not been previously associated to the HCM phenotype. The five non-coding variants were located in the intronic regions very close to acceptor or donor splicing sites, and were found to be heterozygous. No other mutations in the coding regions of screened genes were found in these patients. We first evaluated MAF. None of the five variants were listed in the dbSNP, ExAC, or EVS databases [16,17,18], apart from *SCN5A*-c.393-5C>A and *MYBPC3*-c.906-7G>T, which are recorded in the ExAC database with MAF 0.012% and 0.027%, respectively. Segregation analysis was possible only in two families, but was not informative (Figure S1).

### 2.2. In Silico Analysis

To verify the potential role of the variants identified by genetic screening, we used Alamut Focus version 0.9 (Interactive Biosoftware, Rouen, France). Alamut is a licensed software package available from Interactive Biosoftware (www.interactive-biosoftware.com). To determine the robustness of our in silico predictions, we compared the effect on the splicing process of 10 previously reported intron mutations verified by in vitro/in vivo assay [19,20,21,22,23,24] with the outcome of our Alamut analysis, and obtained similar results (Supplementary Table S1). The results obtained with this predictor software for the novel intron variants are summarized in Table 2.

(1) *MYBPC3*-c.506-2A>C is located in the acceptor splice site of intron 4 of the *MYBPC3* gene. All five algorithms run by Alamut showed that *MYBPC3*-c.506-2A>C severely affected the splicing process. In fact, it caused the loss of the natural acceptor splice site. It also resulted in a cryptic splice site at position *MYBPC3*-c.513.
(2) *MYBPC3*-c.906-7G>T is located in intron 9 of the *MYBPC3* gene. Four algorithms (Splice Site Finder, MaxEnt, GeneSplicer and Human Site Finder) of the Alamut software predicted a small increase in the efficiency of the splicing acceptor site.
(3) *MYBPC3*-c.2308+3G>C: this novel mutation is located in the donor splice site of intron 23 of the *MYBPC3* gene. Two algorithms (MaxEnt and NN Splice) of the Alamut software predicted a consistent alteration with a strength reduction (above 50%) of the natural donor site. The other three algorithms also predicted a donor site alteration, but the percentage of variation induced by the mutation was less than 33% (SSF ≥ −7.1%, GeneSplicer ≥ −32.8%; HSF ≥ −8%). The effect of this change was not predictable by Alamut.
(4) *SCN5A*-c.393-5C>A is located in intron 3 of the SCN5A gene. Two tools of the Alamut software (SSF and HSF) predicted the creation of a novel acceptor site (score increase of more than 70%), although the other three algorithms did not reveal any differences between wild type (WT) and mutated sequences. No algorithm scored the wild-type consensus site.
(5) *ACTC1*-c.617-7T>C: this novel mutation is located in the polypyrimidine tract of the acceptor site of intron 4 in the *ACTC1* gene. Three algorithms (MaxEnt, GeneSplicer and HSF) predicted no differences between WT and mutant; the other two revealed minimal differences.

### 2.3. In Vitro Analysis

**Figure 1:**
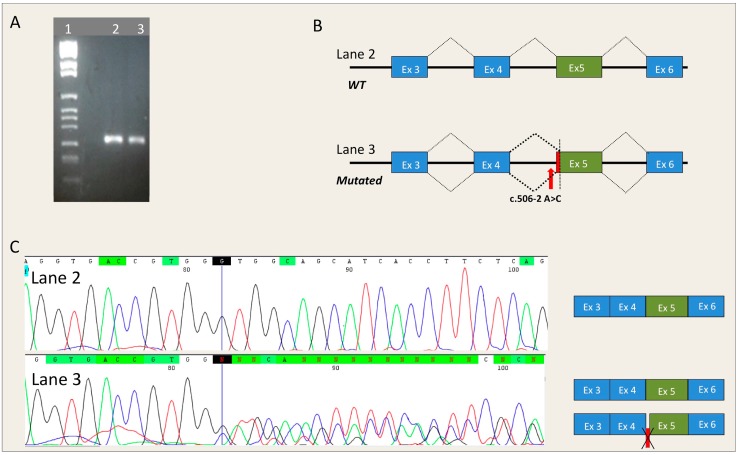
Effect of the *MYBPC3*-c.506-2A>C mutation on the splicing mechanism. (**A**) Electrophoresis of the RT-PCR analysis of mRNA extracted from the peripheral blood of a patient carrying the *MYBPC3*-c.506-2A>C mutation. **Lane 1**: Marker IX (Roche Diagnostics); **Lane 2**: Control; **Lane 3**: Patient carrying the *MYBPC3*-c.506-2A>C mutation; (**B**) Schematic representation of the splicing process in subjects with and without the *MYBPC3*-c.506-2A>C mutation. The red arrow represents the position of the mutation, the red box represents the deletion of seven nucleotides occurring in exon 5 of *MYBPC3*; (**C**) Electropherograms obtained from DNA sequencing of the fragments shown in panel A and relative splicing results (Black, red, green and blue peaks represent the G, T, A, and C bases, respectively). Deletion of seven nucleotides is represented by a red box. The blue line in the electropherograms indicates the end of exon 4 of *MYBPC3* cDNA.

*MYBPC3*-c.506-2A>C. The RNA of an HCM patient carrying mutation *MYBPC3*-c.506-2A>C was extracted from peripheral blood and was retrotranscribed by RT-PCR. The region spanning from exon 3 to exon 6 of *MYBPC3* was amplified by specific primers. The *MYBPC3*-c.506-2A>C mutation causes the complete loss of the canonic acceptor splicing site and the activation of a cryptic 3′ splice site at position c.513, that induces the loss of seven nucleotides of exon 5. This RNA alteration results in the substitution of glycine 169 with an alanine and a subsequent frame-shift and generation of a premature stop codon (*MYBPC3*-G169AfsX14). The patient, who carried the mutation in a heterozygous pattern, showed both a normal and an aberrant transcript (Figure 1).

Since neither the RNA of HCM patients carrying mutation *MYBPC3*-c.2308+3G>C, *MYBPC3*-c.906-7G>T or *ACTC1*-c.617-7T>C, nor the RNA of a BrS patient carrying the *SCN5A*-c.393-5C>A mutation was available, we used a minigene system to verify the results obtained with the Alamut predictor software.

**Figure 2:**
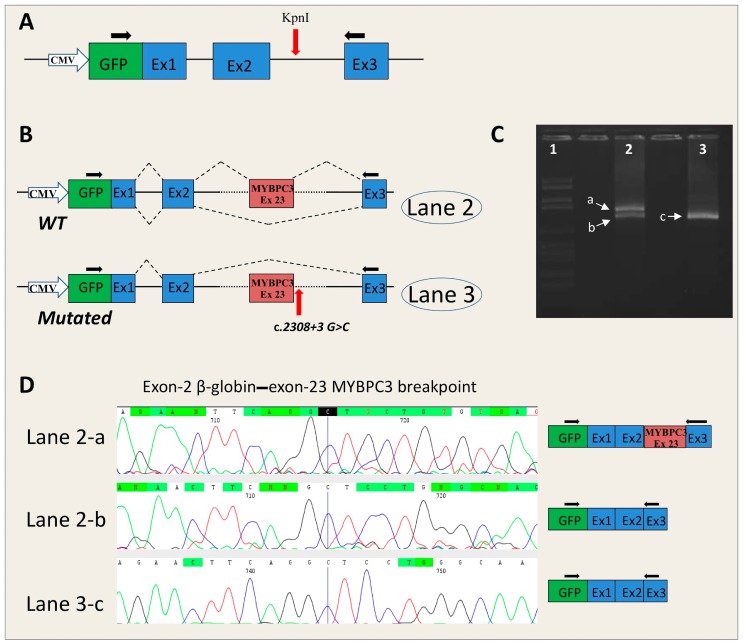
Effect of the *MYBPC3*-c.2308+3G>C mutation on the splicing mechanism. (**A**) Schematic representation of the pMGene empty vector. The GFP coding sequence (green box) is fused with exon 1 of the human β-globin. The exons of human β-globin are shown in blue boxes. The red arrow shows the position of the KpnI restriction site where the insert, containing the exon and the 5′ and 3′ intronic flanking regions, was cloned. The black arrows indicate the primers used for PCR analysis; (**B**) Schematic representation of the splicing process in pMGene-*MYBPC3* constructs (WT, upside; mutated, downside). The red arrow indicates the position of the mutation. The blue boxes represent the exons of human β-globin, the red box represents the *MYBPC3*-exon 23 cloned in the pMGene vector; (**C**) Electrophoresis of the RT-PCR analysis of mRNA extracted from cell lines transfected with pMGene-*MYBPC3* (WT or mutated). **Lane 1**: Marker VI (Roche Diagnostics); **Lane 2**: pMGene-*MYPC3*-WT; **Lane 3**: mutated pMGene-*MYBPC3*; (**D**) Electropherograms obtained from the DNA sequencing of fragments a, b, and c (right) extrapolated from panel B and relative splicing results (Black, red, green and blue peaks represent the G, T, A, and C bases, respectively). The blue line in the electropherograms shows the end of exon 2 of β-globin.

*MYBPC3*-c.2308+3G>C. The analysis of the RNA obtained from the minigene system showed the generation of two transcripts (normal and alternative) in pMGene-*MYBPC3*-WT and only one (alternative) in pMGene-*MYBPC3*-c.2308+3G>C (Figure 2).

The alternative transcript, obtained by minigene assay, arose from the skipping of the *MYBPC3* gene exon 23, which hypothetically is also present in the RNA of the patient, but where possible direct analysis should be performed (Figure 2). This alteration causes the substitution of leucine 717 with a threonine, and premature generation of a stop codon after 51 codons (*MYBPC3*-L717TfsX51).

**Figure 3:**
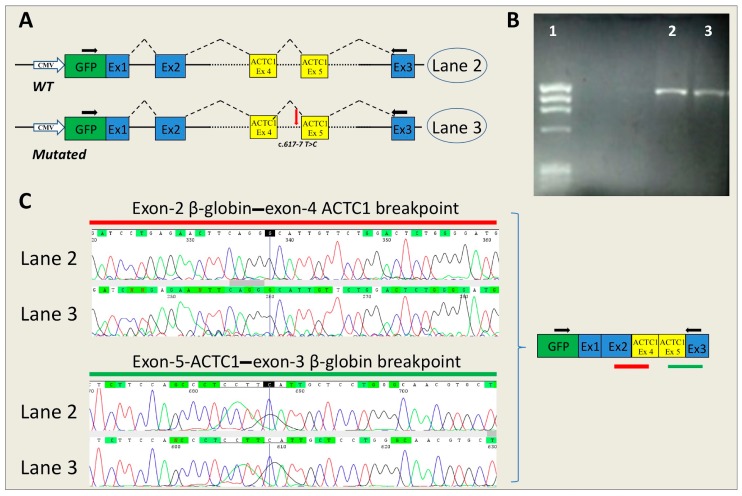
Effect of the *ACTC1*-c.617-7T>C mutation on the splicing mechanism. (**A**) Schematic representation of the splicing process in pMGene-*ACTC1* constructs (WT, upside; mutated, downside). The red arrow shows the position of the mutation. The blue boxes represent the exons of human β-globin, the yellow boxes represent the *ACTC1* exons cloned in the pMGene vector. The black arrows indicate the primers used for PCR analysis; (**B**) RT-PCR analysis of mRNA extracted from cell lines transfected with pMGene-*ACTC1* (WT or mutated). **Lane 1**: Marker IX (Roche Diagnostics); **Lane 2**, pMGene-*ACTC1*-WT; **Lane 3**: mutated pMGene-*ACTC1*; (**C**) Electropherograms obtained from the DNA sequencing of fragments shown in panel B and relative splicing results (Black, red, green and blue peaks represent the G, T, A, and C bases, respectively). The blue lines in the electropherograms show the end of exon 2 of β-globin (upside) and the end of exon 5 of *ACTC1* (downside). The green and red lines show the position of the reported sequences.

*ACTC1*-c.617-7T>C and *MYBPC3*-c.906-7G>T. No RNA alterations were found in the mutated *ACTC1* (Figure 3) or in *MYBPC3* minigenes compared with the WT.

**Figure 4:**
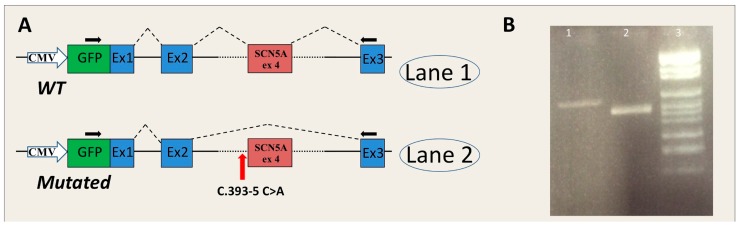

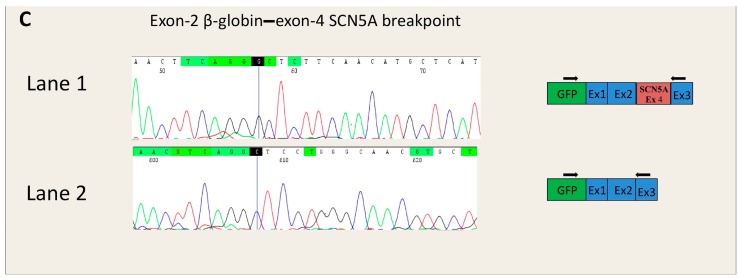
Effect of the *SCN5A*-c.393-5C>A mutation on the splicing mechanism. (**A**) Schematic representation of the splicing process in pMGene-*SCN5A* constructs (WT, upside; mutated, downside). The red arrow shows the position of the mutation. The blue boxes represent the exons of human β-globin, the red box represents the *SCN5A*-exon 4 cloned in the pMGene vector. The black arrows indicate the primers used for PCR analysis; (**B**) RT-PCR analysis of mRNA extracted from cell lines transfected with pMGene-*SCN5A* (WT or mutated). **Lane 1**: pMGene-*SCN5A*-WT; **Lane 2**: mutated pMGene-*SCN5A*; **Lane 3**: Marker VI (Roche Diagnostics); (**C**) Electropherograms obtained from DNA sequencing of the fragments shown in panel B and relative splicing results (Black, red, green and blue peaks represent the G, T, A, and C bases, respectively). The blue lines in the electropherograms show the end of exon 2 of β-globin.

*SCN5A*-c.393-5C>A. The analysis of the RNA obtained from the minigene system showed the generation of an alternative transcript in pMGene-*SCN5A*-c.393-5C>A generated from the skipping of exon 4, which hypothetically is also present in the RNA of the patient. The normal transcript was obtained from the pMGene-*SCN5A*-WT (Figure 4). The skipping of exon 4 causes the in-frame deletion of 30 amino acids in the protein (Na_v_1.5-L132_E161del). The deletion is located in the S1 transmembrane segment of the DI domain [25].

## 3. Discussion

RNA splicing is the process during which the correct recognition of splicing sequences enables intron removal and exon joining. Variations affecting splicing sites impair this process and produce a sizeable percentage of genetic diseases. About 10% of disease-causing mutations annotated in the Human Gene Mutation Database [26] are intronic variations that give rise to mRNA splicing. Intron variants play a crucial role in the etiology of inherited cardiac conditions including cardiomyopathies and ion channelopathies. In this setting, genetic cascade testing is recommended after the initial identification of a pathogenic variation in order to identify asymptomatic relatives who might be at risk of disease-related complications, mainly the risk of sudden cardiac death [27]. Therefore, the identification of a causal variant is essential for a correct diagnosis and to plan other pre-symptomatic clinical decisions. Notably, the Association for Clinical Genetic Science does not recommend predictive testing for a variant of uncertain significant (VUS) for other family members [28].

In this study, we analyzed the pathogenic effect of five intronic variants (*MYBPC3*-c.506-2A>C, *MYBPC3*-c.906-7G>T, *MYBPC3*-c.2308+3G>C, *SCN5A*-c.393-5C>A, and *ACTC1*-c.617-7T>C) that may affect the splicing process. We found these variants in the setting of genetic testing for cardiomyopathies, which involved the analysis of 20 genes (coding regions and their flanking portions), selected on the basis of clinical suspicion. Splicing mutations accounted for 8.1% of all mutations detected, which is comparable to the percentage of splicing mutations annotated in the Human Gene Mutation Database [26]. Apart from *MYBPC3*-c.906-7G>T and *SCN5A*-c.393-5C>A, no variant was recorded in the dbSNP, ExAC, or EVS databases [16,17,18]. Variant *MYBPC3*-c.906-7G>T was identified in a patient affected by HCM; it is present in the ExAC database showing a MAF of 0.027%, which is just above the prevalence of HCM (0.02%). The *SCN5A*-c.393-5C>A variant was identified in a patient with suspected BrS: it had a MAF of 0.012%, which is lower than the allowable estimated disorder allele frequency (prevalence of BrS: 0.05% [29]). Variants with an MAF below cutoff, based on disorder frequency and inheritance pattern, are most likely to be pathogenic [3]. Based on MAF criteria, four of the five intronic variants are pathogenic. Co-segregation analysis of the variant and the disorder within families was possible only for *MYBPC3*-c.506-2A>C and *SCN5A*-c.393-5C>A, but the number of informative meioses within the pedigrees was not sufficient to establish pathogenicity [28]. However, these two variants co-occurred with the disorder (HCM and BrS, respectively) in previous reports [15,30].

Verification of a potential disease-related mutation is performed using in silico procedures and in vitro functional analysis. Notably, results are obtained sooner with the former procedure, whereas in vitro functional analysis can be performed only in well-equipped laboratories. In our study, the mutation disrupting the canonical AG/GT dinucleotides (*MYBPC3*-c.506-2A>C) resulted in a splice defect due to the loss of a canonical site and the use of a cryptic acceptor site 7 nucleotides downstream. In the case of this variant, there was a 100% concordance between the in silico prediction, which highlighted the activation of a cryptic 3′ splice site, and the results of the RNA analysis of the patient’s blood. Bioinformatics analysis of *MYBPC3*-c.2308+3G>C revealed that the score of the canonical donor splice site was decreased using all five bioinformatics tools, and the decrease exceeded 50% with two tools. Accordingly, minigene analysis of the mutated construct revealed skipping of *MYBPC3* exon 23. However, HEK293 cells transfected with the WT minigene showed the coexistence of normal and skipped transcripts, therefore suggesting that the canonical 5′ss of intron 23 is weak and is not always recognized in HEK293 cells.

In silico analysis of *SCN5A*-c.393-5C>A showed a de novo creation of an acceptor site 3 nucleotides upstream the canonical site, whereas the minigene approach revealed skipping of *SCN5A* exon 4. In this case, the creation of a de novo 3′ss, suggested by the in silico analysis, did not correspond to the splicing alteration that occurred in vitro, which is consistent with exon skipping. Of note, the wild-type consensus site was not scored. The better the definition of the consensus site, the more reliable the predictions. If the wild-type consensus site is poorly defined or not defined, the bioinformatics predictions may not reflect the alternative situation. Bioinformatics tools, as well as in vitro analysis conducted with the minigene approach, did not show altered RNA splicing in the presence of variants *MYBPC3*-c.906-7G>T and *ACTC1*-c.617-7T>C.

Splicing alterations elicited by the *MYBPC3*-c.506-2A>C and *MYBPC3*-c.2308+3G>C mutations produced a frame-shift that translates in the loss of function of the mutated allele, possibly due to nonsense-mediated decay or C-terminal truncations; this pathogenetic mechanism is in agreement with the main pathogenic mechanism by which *MYBPC3* mutations act, i.e., the haploinsufficiency. The skipping of exon 4 produced by *SCN5A*-c.393-5C>A causes the in-frame deletion of 30 amino acids in the S1-DI transmembrane segment of the cardiac sodium channel alpha-subunit. In each domain, the S1–S3 segments strongly interact with the S4 segment to form the voltage-sensor domain [25,31]. It is plausible that the lack of 30 amino acids in the S1-DI segment leads to loss of function of the cardiac sodium channel, which is compatible with BrS symptoms. Therefore, our results also shed light on the molecular basis and the likely pathogenic mechanism in two HCM patients/families and in one BrS patient/family.

Finally, the procedure described herein identified three disease-causing mutations out of the five evaluated. Consequently, the three affected families are candidates for cascade genetic screening to identify asymptomatic carriers in the families, who can then be enrolled in treatment or monitoring programs.

## 4. Materials and Methods

### 4.1. Molecular Genetics

Genomic DNA was isolated from peripheral whole blood with the Nucleon BACC2 kit (GE Healthcare, Life Sciences, Little Chalfont Buckinghamshire, UK). All coding exons, and 5′ and 3′ UTRs of genes involved in inherited cardiomyopathies associated to sudden death were amplified by PCR and analyzed by automatic sequencing using previously reported protocols [32]. Based on diagnostic suspicion, patients were screened for a specific gene panel (Table 1). In some patients, we analyzed a large number of target genes using Next Generation Sequencing methodology and the variant selection criteria reported previously [33]. Informed consent to perform genetic analysis was obtained from patients according to the second Helsinki Declaration [34]. Genetic analysis was extended to relatives when possible to verify segregation of a putative mutation with disease in the family.

### 4.2. Splice-Site Prediction Analysis

Alamut software was used for in silico prediction of splice-affecting nucleotide variants. Genomic sequences (WT and mutant) were processed by this predictor software using five splicing prediction tools (SpliceSiteFinder-like, MaxEntScan, Neural Network Splice, GeneSplicer, and Human Splicing Finder). Each tool is based on different algorithms: SpliceSiteFinder-like uses position weight matrices computed from a set of human constitutive exon/intron junctions for donor and acceptor sites; MaxEntScan and NNPLICE are based on the Maximum Entropy principle and neural networks, respectively. GeneSplicer combines several splice site detection techniques, namely Markov models; and HSF is based on position weight matrices with some position-dependent logic [35].

### 4.3. In Vitro RNA Splicing Analysis of MYBPC3-c.506-2A>C

Total RNA was directly extracted from 5 mL of patients’ peripheral blood by Trizol Reagent (Thermo Fischer Scientific, Waltham, MA, USA). RNA retro-transcription was performed as previously described [9], using specific primers spanning from exon 3 to exon 6. Forward primer was drafted to overlap between exon 3 and 4 of the *MYBPC3* transcript (Forward, 5′-CAAGTCCCAAAGGGTCAAGCTC-3′ and Reverse, 5′-GTGGACACCTCACAGCGGTA-3′).

### 4.4. In Vitro RNA Splicing Analysis by Minigene

#### 4.4.1. Insert Generation (*MYBPC3*-c.906-7G>T, *MYBPC3*-c.2308+3G>C, *SCN5A*-c.393-5C>A, *ACTC1*-c.617-7T>C)

The genomic regions affected by non-coding mutations were amplified by PCR using PCR Master Mix (Promega, Madison, WI, USA). The PCR amplicons contained both the interested exon (the region including exon 9-10-11 of *MYBPC3* gene; exon 23-*MYBPC3*; exon 4-*SCN5A*; the region spanning from exon 4 to exon 5-*ACTC1*) and the 5′ and 3′ intronic flanking regions. The primers contained a KpnI restriction sequence at the 5′ terminus. The primers used for PCR are listed in Supplementary data (Table S2). The sizes of inserts obtained by PCR were approximately 1000 base pairs.

#### 4.4.2. Minigene Plasmid Construction, Expression, and Transcripts Analysis

The PCR fragments obtained by PCR were cloned into the pMGene vector [11]. The empty vector contained the human β-globin gene, from the starting codon to the stop codon (including introns), and the GFP coding sequence fused with exon 1. A unique KpnI restriction site was located in the middle of β-globin intron 2 (Figure 2A). PCR products and the pMGgene vector were digested by the KpnI restriction enzyme. The open pMGene vector was then dephosphorylated. The digested inserts were then cloned into the pMGene vector using the LigaFast Rapid DNA Ligation System (Promega). All clones were sequenced, and the WT and mutated forms of each construct (pMG-*MYBPC3*, pMG-*ACTC1*, and pMG-*SCN5A*) were selected for expression experiments.

HEK-293 cells were grown in Dulbecco’s modified eagle medium supplemented with 10% FBS, 2 mM l-glutamine and 1% penicillin/streptomycin in a humidified, 5% CO_2_ atmosphere at 37 °C. Cells were transiently transfected with 2 µg of WT or mutated pMGene using FuGene HD (Promega). Forty-eight hours after transfection, cells were collected and RNA was extracted by Trizol Reagent (Life Technologies). RNA retrotranscription was performed by SuperScript VILO (Life Technologies) starting from 1 µg of total RNA and using random primers. The cDNA obtained was amplified with the following primers: forward, 5′-ACGACGGCAACTACAAGACC-3′ and reverse, 5′-CACACCAGCCACCACTTTC-3′ annealing in the GFP-coding region and in exon 3 of β-globin, respectively (Figure 2A). Since the forward primer annealed in the GFP-coding region, only located in the transcripts derived from the plasmid construct, the PCR did not select endogenous β-globin, *MYBPC3*, *ACTC1*, or *SCN5A* transcripts.

## 5. Conclusions

This study illustrates the efficacy of an integrated approach, constituted by indirect criteria (MAF, co-segregation, and co-occurrence), in silico simulation and RNA analysis (directly or with minigene technology) to determine the effect of potential splice site variants. Bioinformatics can be used to filter more likely disease-causing variants from among the large number of candidates detected by high-throughput DNA sequencing. Subsequently, in silico predictions, particularly when obtained by the evaluation of multiple algorithms, can be verified in vitro. Notably, the RNA analysis performed by RT-PCR of the patient’s RNA or by the minigene approach should invariably be used whenever possible to reach a reliable conclusion concerning pathogenicity.

In conclusion, we identified the molecular basis and the likely pathogenic mechanism in two HCM patients/families and in one BrS patient/family. These findings can improve the genetic counselling of patients and their relatives.

## Figures and Tables

**Table 1:** Panel of genes screened according to diagnostic suspicion.

Gene	Protein	Pathological Phenotype
*MYH7*	Myosin, heavy chain 7	HCM, DCM
*MYBPC3*	Myosin binding protein C	HCM, DCM
*TNNT2*	Troponin T2, cardiac type	HCM, DCM
*TNNI3*	Troponin I3, cardiac type	HCM
*TPM1*	Tropomyosin 1	HCM
*MYL2*	Myosin, light polypeptide 2	HCM
*MYL3*	Myosin, light polypeptide 3	HCM
*ACTC1*	Actin, alpha, cardiac muscle 1	HCM
*LMNA A/C*	Lamin A/C	DCM
*SCN5A*	Voltage-gated sodium channel α-subunit	BrS, LQTS, DCM
*KCNQ1*	Voltage-gated potassium channel α-subunit	LQTS
*KCNH2*	Voltage-gated potassium channel α-subunit	LQTS
*KNCE1*	Potassium voltage-gated channel subfamily E member 1 (β subunit)	LQTS
*KCNE2*	Potassium voltage-gated channel subfamily E member 2 (β subunit)	LQTS
*DSP*	Desmoplakin	ARVC
*PKP2*	Plakophilin 2	ARVC
*DSG2*	Desmoglein 2	ARVC
*DSC2*	Desmocollin 2	ARVC
*JUP*	Junction plakoglobin	ARVC
*RYR2*	Ryanodine receptor 2	CPVT

HCM, Hypertrophic cardiomyopathy; DCM, Dilated cardiomyopathy; LQTS, Long QT syndrome; BrS, Brugada syndrome; ARVC, Arrhythmogenic right ventricular cardiomyopathy; CPVT, Catecholaminergic polymorphic ventricular tachycardia.

**Table 2:** Splicing analysis results obtained with five algorithms and the Alamut software.

Gene	Nucleotide Variation	cDNA Position ^§^	Splice Site Finder (0–100)		Max Ent Scan (0–16)		NNSPLICE (0–1)		Gene Splicer (0–15)		Human Splicing Finder (0–100)		Alamut Predicted Change
			WT	MUT	WT	MUT	WT	MUT	WT	MUT	WT	MUT	
*MYBPC3*	c.506-2A>C	c.506 *	87.86	―	12.9	―	0.96	―	10.25	―	92.79	―	Acceptor splice site: −100%
		c.513 **	―	75.34	―	4.64	NE	NE	―	3.65	77.42	79.45	
*MYBPC3*	c.906-7G>T	c.906 *	73.61	78.66	3.82	4.44	NE	NE	__	2.29	80.55	82.75	Acceptor splice site: +223%
*MYBPC3*	c.2308+3G>C	c.2308 *	77.58	72.06	8.99	3.77	0.87	―	12.67	8.52	85.13	78.32	Donor splice site: −52%
*ACTC1*	c.617-7T>C	c.617 *	84.72	83.21	6.55	6.71	0.92	0.83	8.24	8.54	83.31	83.73	Acceptor splice site: −2%
*SCN5A*	c.393-5C>A	c.393-3	―	70.16	―	0.85	NE	NE	NE	NE	―	77.83	Acceptor splice site: 0%

Parentheses show the score range for each algorithm; ―, splice site not detected; NE, splice site not evaluated by the algorithm; WT: wild type sequence; MUT: mutated sequence; NNSPLICE: Neural Network Splice ; ^§^ first nucleotide of the splice site; * natural splice site; ** cryptic splice site.

## Supplementary Materials

Supplementary materials can be found at www.mdpi.com/1422-0067/17/11/1883/s1.
