# Supplementary material for: Functional Studies and In Silico Analyses to Evaluate Non-Coding Variants in Inherited Cardiomyopathies
Source: Int J Mol Sci. 2016 Nov 10;17(11):1883. doi: 10.3390/ijms17111883 (PMC5133883; doi:10.3390/ijms17111883)
Supplement: Supplementary file 1 [file ijms-17-01883-s001.pdf]

Supplementary Materials: Functional Studies and In Silico Analyses to Evaluate Non-Coding Variants in Inherited Cardiomyopathies

Giulia Frisso, Nicola Detta, Pamela Coppola, Cristina Mazzaccara, Maria Rosaria Pricolo, Antonio D'Onofrio, Giuseppe Limongelli, Raffaele Calabrò and Francesco Salvatore

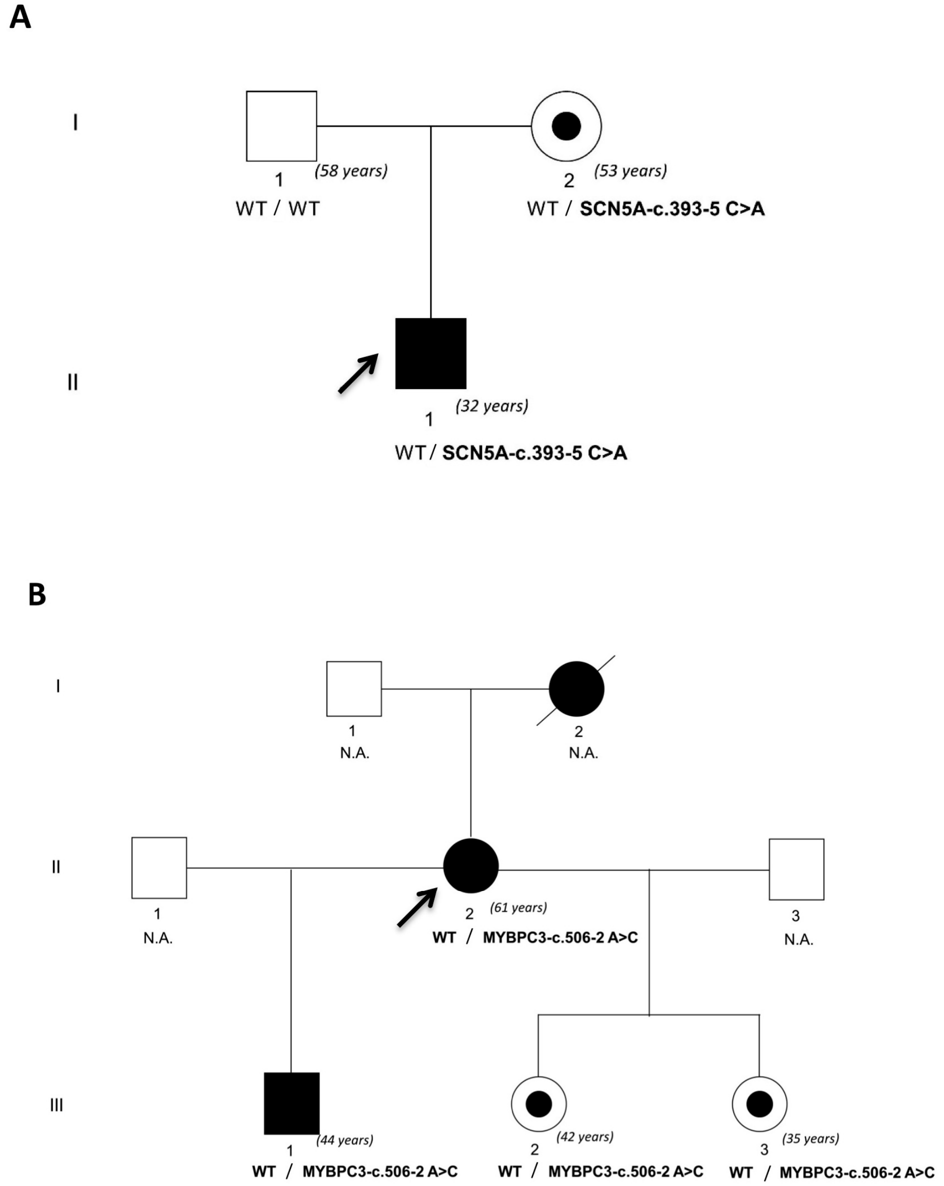

Figure S1. Pedigrees of the family with Brugada syndrome (**A**) or hypertrophic cardiomyopathy (**B**). Open symbols represent subjects with a negative phenotype. Black symbols represent clinically affected subjects. Circles with solid centers indicate unaffected female mutation carriers. The diagonal line indicates a deceased family member. The arrows indicate the proband. The ages of subjects are reported in brackets. N.A.: not analyzed; WT: wild type.

Table S1. Effect on the splicing process of 10 previously reported intron mutations, verified by in vitro/in vivo assay, compared with the outcome of Alamut analysis. Parentheses show the score range for each algorithm; —, the splice site is not detected; NE, splice site not evaluated by the algorithm; §, first nucleotide of the splice site; * natural splice site; # effect verified on patient's mRNA; ## effect verified on minigene construct. WT: wild type sequence; MUT: mutated sequence.

Gene	Nucleotide Variation	cDNA Position §	Splice Site Finder (0–100)		Max Ent Scan (0–16)		NNSPLICE (0–1)		Gene Splicer (0–15)		Human Splicing Finder (0–100)		In Vitro Splicing Studies	Alamut Predicted Change
			WT	MUT	WT	MUT	WT	MUT	WT	MUT	WT	MUT		
MYBPC3	c.821+5G>A	c.821 *	82.33	70.18	9.30	—	0.94	—	10.87	—	87.83	75.66	exon 7 skipped #	Donor splice site: −67%
MYBPC3	c.927-9G>A	c927 *	NE	NE	NE	NE	NE	NE	NE	NE	81.91	81.79	exon 11 skipped ##	Acceptor splice site: −26%
MYBPC3	c.1624+4A>T	c.1624 *	80.59	70.42	7.75	3.55	0.90	—	9.87	3.17	90.86	82.05	exon 17 skipped #	Donor splice site: −51%
MYBPC3	c.1928-2A>G	c.1928 *	89.30	—	9.92	—	0.75	—	13.48	—	89.60	—	inclusion intron 20 #	Acceptor splice site: −100% Skipping of exon 21 very likely
MYBPC3	c.3190+5G>A	c.3190 *	72.21	—	6.18	—	NE	NE	6.34	1.42	83.30	71.14	exon 29 skipped ##	Donor splice site: −71%
SCN5A	c.1140+1G>A	c.1140 *	85.46	—	6.99	—	0.90	—	5.10	—	90.04	—	exon 9 skipped ##	Donor splice site: −100% Skipping of exon 9 very likely
KCNQ1	c.477+5G>A	c.477 *	80.40	—	9.89	4.52	0.97	—	11.44	6.41	85.49	73.33	use of a cryptic 5′ss c.477+80 ##	Donor splice site: −48%
KCNQ1	c.478-2A>T	c.478 *	90.02	—	11.78	—	0.83	—	9.23	—	94.27	—	exon 3 skipped #	Acceptor splice site: −100% Skipping of exon 3 very likely
KCNQ1	c.1032+5G>A	c.1032 *	77.95	—	9.00	3.46	0.97	—	13.86	7.74	85.15	72.98	exon 7 skipped ##	Donor splice site: −47%
TNNT2	c.821+1G>A	c.821 *	78.12	—	8.46	—	0.99	—	6.85	—	83.32	—	exon 15 skipped #	Donor splice site: −100% Skipping of exon 15 very likely

Table S2. List of primers used to amplify the target genomic sequences of inserts that will be cloned in the pMG vector.

Gene	Primer Direction	KpnI Tail (Uppercase)	Primer Sequence (5′–3′) (Lowercase)
MYBPC3	Forward	CGGGGTACC	cctggctcccttcatccta
	Reverse	CGGGGTACC	cacccccagatccaaagag
ACTC2	Forward	CGGGGTACC	gcatccccaaggagaataca
	Reverse	CGGGGTACC	ccctttaatgagccatcagg
SCN5A	Forward	CGGGGTACC	taccagaaaggcaggacagg
	Reverse	CGGGGTACC	ttaggcaggacagggagaaa
